# The Organon, Section 153

**Published:** 1881-01

**Authors:** P. P. Wells

**Affiliations:** Brooklyn, N. Y.


### THE ORGANON, SECTION 153.

BY P. P. WELLS, M. D., BROOKLYN, N. Y.

In Sect. 18 of this much neglected book, we read "that the totality of the symptoms is the sole indication for the selection of remedies." This is true if we understand that in this totality are contained the symptoms which control the choice. It is not true if by this be meant that all the symptoms in this totality are of equal authority in their control of this choice. That this is not what the author intended to teach is made quite plain by Sect. 153, which may be taken as a commentary on Sect. 18. In this, Sect. 153, he says, "In searching after the specific remedy *** we ought to be particularly and almost exclusively attentive to the symptoms that are *striking*, *singular*, *extraordinary* and *peculiar*, (characteristic) *for it is to these latter that similar symptons, from among those created by the medicine, ought to correspond*, in order to constitute it the remedy most suitable to the cure. On the other hand the more vague and general symptoms *** merit little attention, because almost all diseases and medicines produce something as general."

Now, in seeking for the specific remedy for a given case of sickness according to the Homœopathic method, a right understanding of Sections 18 and 153 is indispensable, if mistake and failure are to be avoided. To find all the symptoms of the case to be treated in a single remedy is often impossible, for the reason they are not in the record of any one, so to seek for them will often be only labor lost. Natural diseases are not gotten up in patterns exactly adapted to those recorded as the result of the action of ingested drugs. The "like" which cures does not necessarily consist in this resemblance in its *intirety*. When this does obtain and can be found, the cure is for this reason the more certainly assured. But if cures were limited to such cases the practical value of the

Homœopathic law would be reduced far below its true standard.

How then are we to reconcile these two sections when we accept them as our practical guides? The one requires the *totality* of the symptoms, the other, those most *striking*, etc. The one seems to demand the *whole*, the other but a *part*. We answer, Sect. 18 teaches simply this, that we have no other guides to the selection of curatives than the symptoms of the case to be cured. Its chief intent is to exclude from this selection all abstract notions and hypotheses of whatever name. This was the more needed at the time this paragraph was written, for the reason that these then constituted almost the entire furnishing of the then current school of medical practice. It is still needed for the reason that the old time poverty in practical resources is still prevalent, as is the old endeavor to conceal this fact by pretences to knowledge of that which only exists in the imagination, which pretences are not more respectable because presented in terms which time and teaching have incorporated into current medical thought and practice.

"The symptoms *alone* the guide!" says the objector. That is just what this paragraph is intended to teach, and not that every symptom of a case is to be found in the record of its curative before it can be accepted as such. "Then," continues the objector (*old school*) "you treat only symptoms, and not *diseases* at all." This has been cast at the Homeopathic school as a reproach, from the beginning; and with as much of boldness and arrogance as if its opponents had really something else to treat. "We treat diseases." Indeed! What are these but names, often arbitrary and without significance, of which nothing is or can be known, except through manifestations to the patient or physician which we call symptoms? Aside from these diseases are, as to all knowledge of them, but abstract ideas of things unknown, and except through these manifestations, unknowable, as objects of curative endeavor. The old school pretence that it treats diseases, as something distinct from these, resolves itself into the very empty abstractions and hypotheses which this eighteenth section was intended to antagonize.

But if the symptoms are the only guides to the selection of the curative remedy, what becomes of the vaunted pathology of which we hear so much, and so often from those who are slightly informed as to its nature, place or importance in our practical duties. To guard against the *wrong use* of this valuable science was another occasion for giving us this eighteenth section. To put it as a teacher in the selection of curatives, to the exclusion of the symptoms from that function, is to put it where it has no place in a rational system of healing, certainly none under the control of a natural law, which discloses the curative relationship as existing in the similarity between the symptoms of the drug and the disease. Where, then, is the practical use of this so highly prized science of pathology? In the duty of prescribing for the sick, its use is limited to aiding a right understanding of the nature and value of the symptoms revealed in the case in hand. Beyond this it has no function in the process of prescribing. Pathology, to illustrate, teaches a difference between inflammations and neuralgias. Both are attended by pains of the severest kind, but this science teaches that these have a different significance and often different importance, as the case in hand belongs to one class or the other. A knowledge of the science of pathology will enable us to relegate our case to its proper class, and there its function ceases. It can not go beyond this; and having decided the case a neuralgia, say the remedy is *Aconite* or *Bell.* or *Bry.* or *Colocyn.* or *Hyosc.* or *Lach.* or *Merc.* or *Nux* or *Puls.* or *Rhus* or *Spig.* or either of the other many remedies which a given case may demand for its cure under the law. To attempt to give to this science this decision is to inpose on it a function wholly out of the sphere of its legitimate use. This is guarded against by the wise direction of the eighteenth section.

The one hundred and fifty-third section of the *Organon*, if taken as a commentary on the eighteenth, plainly indicates the above as the true intent of the author of that section. The direction to have in our search for the specific curative, chief reference to those symptoms which are *striking, extraordinary and peculiar*, paying but slight regard comparatively, to those more common and general, confirms this perfectly.

But how shall we understand the terms of the commentary? What by the words *striking*, *extraordinary*, etc? Our first remark in our endeavor to get at the true meaning of these is that by the "*most striking*," the author can not mean that symptom which first and most forcibly seizes the attention of the physician, the patient and his friends. To make this apparent take a case of dysentery. That which first arrests and holds the attention of all is the pain and tenesmus. But these are so general that they belong to all cases of this disease, and therefore by this fact are relegated to that category of symptoms which the author assures us "merit little attention." Without these no case is dysentery. It is evident, then, the author does not use the word in this sense. Its selection seems less felicitous than is common with him, and has led often to a wrong conclusion as to the importance of these general, or defining, symptoms in the search for specific remedies according to the requirements of the practical medicine he taught. That that is the most "striking," which is the most painful and intrusive on the attention, has been the understanding of this direction, and this has led to giving to these general symptoms just the consideration which the author tells us they do not merit. His real meaning is better expressed by the last term employed to indicate the class of symptoms to be chiefly regarded in our search. "Peculiar." This is it. But what does he mean by the word here? Evidently that we are to give chief attention to symptoms which are "peculiar" to the case in hand. Not necessarily to those which cause the patient most suffering. That which is peculiar to the case characterizes it as a member of a family. The general, or defining, symptoms declare the family to which this member belongs. Then it is the peculiar, or specific symptoms which are our chief guides in our discovery of the specific cure of the case. But it may be asked, is not that peculiar to a disease which is found in each example of it? In a certain sense it is; but not in that in which it is used here. If this were so, then in a case of dysentery, for example, we should have, under this direction, only to notice the pain, tenesmus, and the other defining symptoms which belong to this, and all other cases of the class, and find in the similar of these the curative under the law. We have all tried this, and have been disappointed in our expectations of the cure we supposed the law promised as the result of this proceeding. The disappointment came from our misunderstanding of the requirements of the law. It will come in every case so treated. Success can follow only in those where the remedy chosen happened to have in its record, with those defining symptoms, those other and less obtrusive ones which individualize the case, and in which curative relationship between drugs and diseases alone resides. If the cure follows in cases so treated, in the prompt and pleasant manner a right compliance with the demands of the law assures, it is because the practitioner has been guilty of a fortunate blunder. This will be sufficiently plain if we remember that *Homœeopathic* prescribing is *specific* prescribing. That is, finding and giving to the sick the one specific medicine the cure of his case requires under the law. Homœopathy presumes the existence of such a remedy in every case of sickness. It imposes on the physician the duty of finding it. If in any case, as may well happen, either from poverty of our resources, or from lack of knowledge, the one remedy can not be found, *i. e.*, a remedy which in its known effects on the organism are found the symptoms which constitute it the specific in the case, by virtue of the required similarity, then that must be selected which has greater similarity to the elements of the diseased manifestation than any other. This resort to that which is less than perfect, because of the above necessity, is no argument against the right of the presumption of the existence of that which is perfect, *i. e.*, some drug in which is the power to produce symptoms with the required resemblance to constitute it the required specific. This drug may not yet have been proved, or if proved, not known to the physician, and hence the necessity of this resort to that which is less than perfect. Neither does the fact that this resort is followed at times by a cure, which though less prompt and complete than that from a specific remedy, is nevertheless ultimately a *cure*, excuse the prescriber from the utmost endeavor to find that which is perfect. This is ever to be the one object of his life work, to find the *one* specific; failing in this endeavor is failing in the first and most important of his duties. Success in this is that which gives brightest joy to the life of the physician. The fruits of this success are the glories which crown the immortal discoverer of specific prescribing, which when he had found, he called *Homœopathy*.

That the above view of the one hundred and fifty-third section which refers defining symptoms to a subordinate importance, in the search for the specific remedy, is the true one, may be seen still more clearly, if we attempt a prescription based on these as a chief guide. The impracticability of this will appear if, when we accept these as our guides, we remember that one remedy is the only specific for our case, and this, that it may be such it must be in its effects on the organism that which is most like those defining symptoms of our case. The case is dysentery, the defining symptoms of which are—*frequent discharges from the rectum of blood or mucus or both, with colic pains, tenesmus and fever.* Now there are, as to the first of these, a multitude of cases met with in practice, the discharges of which are so much alike, and so like those recorded as having resulted from the action of a multitude of remedies on the organism, that no man can tell from these, in a given case, which of this multitude in this particular, is more like the case in hand than the others, and therefore is for this case its specific cure. The discharges are small, of mucus mixed with blood, and here is all they have to tell in very many cases, and the records of the effects of many drugs tell the same story so exactly that no man can tell which of them has most resemblance to that of the case in hand. The same is true of the pains. From these alone no man can tell whether they are more like those which have resulted from one or other of the many drugs from which we have to choose in treating our case, and therefore we can not tell whether one or the other is most like the pain in the case for which he s seeking a remedy. This he may feel sure of, that that which he seeks is one of the many, but which of these? To answer this question on a better foundation than a guess, will necessitate a reference to other elements of the case, and these belong evidently to that other class of symptoms which this one hundred and fifty-third section commends to our chief attention—those which are the specifics of the case. These remarks are equally applicable to the other defining symptoms—the tenesmus and fever.

We have said Homœopathy is specific prescribing. Its practice is ever and only a successive finding of the one specific remedy for each succeeding case as it becomes a subject for treatment. This being found it needs no second for its aid in the cure. If otherwise, then it fails to fulfill the office of a specific, and this is proof sufficient that in this case, at least, the prescriber has failed in his duty as a Homœopathic physician. He has not found the true specific for his case, which, as a representative of this school he was bound to do, and failing in which, so far as this case is concerned, he *quo ad hoc*, ceases to represent that school. If it be true that there are such specifics for the cure of the sick, and that the finding of them is possible, under the guidance of the two sections of the *Organon* we have been discussing, then the superfluity, to saythe least, of all so called adjuvants is demonstrated, whether these be of external or internal application. But we may go further, and as no man can tell beforehand how this so called adjuvant is or is not to modify the action of the specific remedy, that while it is in all cases needless, in many it must by such modifications become positively injurious. This view, it will be seen, effectually disposes of the liberty which has of late, so often and so earnestly, claimed to do as one pleases in the discharge of his practical duties, in this matter of adjuvants, and in all others at variance with the teaching of the two sections we have been considering. If one claims this liberty, and acts upon it in his clinical duties, to the prejudice of the action of the specific remedy selected, then there is another liberty, which, by so doing, he deprives himself of—the liberty to call himself afterward a Homœopathic physician.

The view of practical law and duty which we have been presenting, if admitted as authoritative, will also dispose of another fashion of practice (we can not regard it as aught but a fashion), that of prescribing at the same time two or more remedies to be given in alternation, at definite intervals of time, in the absence of all knowledge of what will be the condition of the patient at the lapse of either of these intervals, and therefore not knowing whether either of the given remedies will or will not be a specific for his case at the time it is directed to be given. If either of the prescribed remedies be the specific for the case in hand the other cannot be. The idea of a specific for a given case, made such by the law of similars, excludes the possibility of a second in the same case, as it is impossible that each of the two can be "*most like.*" One or neither of the two may be, but both cannot. That which is not is at least useless, often mischievous, and never Homœopathic, if to be this it is indispensable that each prescribed medicine shall be that which in its ascertained action on the living organism presents the most perfect likeness to the phenomena of the disease to be cured.

